# Prognostic value of tumor necrosis based on the evaluation of frequency in invasive breast cancer

**DOI:** 10.1186/s12885-023-10943-x

**Published:** 2023-06-09

**Authors:** Jianhua Chen, Zhijun Li, Zhonghua Han, Deyong Kang, Jianli Ma, Yu Yi, Fangmeng Fu, Wenhui Guo, Liqin Zheng, Gangqin Xi, Jiajia He, Lida Qiu, Lianhuang Li, Qingyuan Zhang, Chuan Wang, Jianxin Chen

**Affiliations:** 1 Key Laboratory of OptoElectronic Science and Technology for Medicine of Ministry of Education, Fujian Provincial Key Laboratory of Photonics Technology, College of Photonic and Electronic Engineering, Fujian Normal University, Fuzhou, 350117 China; 2 College of Life Science, Fujian Normal University, Fuzhou, 350117 China; 3 Department of Breast Surgery, Fujian Medical University Union Hospital, Fuzhou, 350001 China; 4 Department of Pathology, Fujian Medical University Union Hospital, Fuzhou, 350001 China; 5 Department of Radiation Oncology, Harbin Medical University Cancer Hospital, Harbin, 150081 China; 6 College of Physics and Electronic Information Engineering, Minjiang University, Fuzhou, 350108 China; 7 Department of Medical Oncology, Harbin Medical University Cancer Hospital, Harbin, 150081 China

**Keywords:** Frequency, Invasive breast cancer, Multiphoton imaging, Tumor necrosis, Spatial heterogeneity, Prognosis

## Abstract

**Background:**

Tumor necrosis (TN) was associated with poor prognosis. However, the traditional classification of TN ignored spatial intratumor heterogeneity, which may be associated with important prognosis. The purpose of this study was to propose a new method to reveal the hidden prognostic value of spatial heterogeneity of TN in invasive breast cancer (IBC).

**Methods:**

Multiphoton microscopy (MPM) was used to obtain multiphoton images from 471 patients. According to the relative spatial positions of TN, tumor cells, collagen fibers and myoepithelium, four spatial heterogeneities of TN (TN1-4) were defined. Based on the frequency of individual TN, TN-score was obtained to investigate the prognostic value of TN.

**Results:**

Patients with high-risk TN had worse 5-year disease-free survival (DFS) than patients with no necrosis (32.5% vs. 64.7%; *P* < 0.0001 in training set; 45.8% vs. 70.8%; *P* = 0.017 in validation set), while patients with low-risk TN had a 5-year DFS comparable to patients with no necrosis (60.0% vs. 64.7%; *P* = 0.497 in training set; 59.8% vs. 70.8%; *P* = 0.121 in validation set). Furthermore, high-risk TN “up-staged” the patients with IBC. Patients with high-risk TN and stage I tumors had a 5-year DFS comparable to patients with stage II tumors (55.6% vs. 62.0%; *P* = 0.565 in training set; 62.5% vs. 66.3%; *P* = 0.856 in validation set), as well as patients with high-risk TN and stage II tumors had a 5-year DFS comparable to patients with stage III tumors (33.3% vs. 24.6%; *P* = 0.271 in training set; 44.4% vs. 39.3%; *P* = 0.519 in validation set).

**Conclusions:**

TN-score was an independent prognostic factor for 5-year DFS. Only high-risk TN was associated with poor prognosis. High-risk TN “up-staged” the patients with IBC. Incorporating TN-score into staging category could improve its performance to stratify patients.

**Supplementary Information:**

The online version contains supplementary material available at 10.1186/s12885-023-10943-x.

## Background

Tumor necrosis (TN) is histologically characterized by homogeneous clusters and sheets of dead and degraded tumor cells that coalesce into an amorphous coagulum, which are admixed with nuclear and cytoplasmic debris [1-3]. It is a common histological feature of solid tumors, and its prognostic significance has been studied in several solid tumors [1, 2, 4-9]. In these studies, classifications based on a presence/absence basis and extent basis were traditionally applied, and the association between TN and poor outcome was found. As a kind of solid tumor, breast cancer was the most commonly diagnosed cancer [10]. The relationship between TN and invasive breast cancer (IBC) outcome has also been widely studied through these two necrotic classification methods. However, there were several conflicting results about the impact of TN on IBC prognosis [11-21]. Based on presence/absence classification, Gilchrist et al. found that the presence of TN was significantly associated with aggressive tumor characteristics including age, lymph nodes and tumor size. The presence of TN was an independent predictor for early recurrence (0 to 2 years after diagnosis) and for survival in the overall 10-year follow-up period [13]. Being consistent with this result, Vincent-Salomon et al. also believed that the presence of TN was an independent predictor for overall survival and metastasis-free survival [16]. Although other studies also reported that TN could predict survival, they believed that this effect was not independent of other high-risk pathological characteristics. In these studies, univariate analysis showed that the presence of necrosis was significantly related to worse survival, but on multivariate analysis with the Cox proportional hazard regression model after filtering competitive risk factors, TN was not an independent predictor of survival [19-21]. What's more, some studies even failed to find a direct association between TN and poor prognosis [14, 20]. These conflicting reports might be attributed to the simple presence/absence binary classification, which ignored intratumor heterogeneity and thus lost some prognostic information [22]. Therefore, Klatte et al. evaluated necrosis as a continuous variable and found that the extent-based classification offered more sensitive risk stratification for renal- or transitional-cell malignancy [22]. However, this risk stratification result, offered by extent-based classification, was inconsistent in the prognosis study of IBC. For example, Fisher et al. classified the necrosis (slight, moderate and marked) into three variables in IBC and found that patients with slight or moderate degrees of necrosis had better DFS than those with marked necrosis [15]. Surprisingly, Leek et al. also classified the necrosis (focal areas of necrosis, widespread areas of necrosis and virtually all necrosis) into three variables in IBC, but they did not observe that the presence of necrosis or high levels of necrosis had a significant impact on either relapse-free or overall survival [11]. Therefore, in IBC, whether TN was an independent prognostic factor remains ill-defined, and a new classification method that could reflect TN heterogeneity was needed clinically. In addition, Wei et al. found that in hepatocellular carcinoma, the presence of necrosis on pathology essentially “up-staged” a patient relative to long-term prognosis [6]. As one of the most common features observed on histopathological examination of solid tumors, the impact of TN on staging category in IBC has not been reported. Staging category was one of the best-established prognostic factors for breast cancer [21]. Clarifying the impact of necrosis on survival and staging category would help stratify patients with IBC.

Multiphoton microscopy (MPM) was a novel optical tool for femtosecond laser as a light source to explore the structure and function of biological samples, especially of tissues. Using the same excitation light, MPM could identify different types of signals: two-photon excited fluorescence (TPEF) and second harmonic generation (SHG), which was well-suitable for the visualization of tumor microenvironment [23]. Cells contained intrinsic fluorophores, such as nicotinamide adenine dinucleotide (NADH) and flavin adenine dinucleotide (FAD), which carried the information of cellular metabolism and generated TPEF signals with the peaks at around 470 nm and 540 nm [24, 25]. Normal cells, precancer cells and cancer cells had different redox ratios (i.e., the ratio of NADH over FAD fluorescence), and they could display different emission spectra under the same excitation [24]. By capturing the TPEF signal, the location of these cells could be displayed on the TPEF images without labeling. SHG could recognize molecules with non-centrosymmetric structures via interaction with incident light. In this way, the intrinsic properties of molecules could be revealed without labeling [26]. Collagen fibers had a non-centrosymmetric molecular structure that could be excited to generate second-harmonic generation (SHG) signals so that their morphology could be directly visualized in situ by SHG imaging [27, 28]. Because the relative spatial distributions of cells and collagen fibers in the tumor microenvironment could be well displayed in the MPM images, in our previous studies, we focused on the location distribution of collagen fibers and tumor cells and observed eight large-scale tumor-associated collagen signatures (TACS1-8) in MPM images [29]. In addition, according to the relative positions between tumor-infiltrating lymphocytes (TILs), tumor cells and collagen fibers, we redefined TILs as TILs-1, TILs-2 and TILs-3 from MPM images [30]. They showed better predictive performance than traditional clinical models, suggesting that the diversity of spatial heterogeneity in tumor microenvironment does contain a lot of prognostic information.

**Fig. 1:**
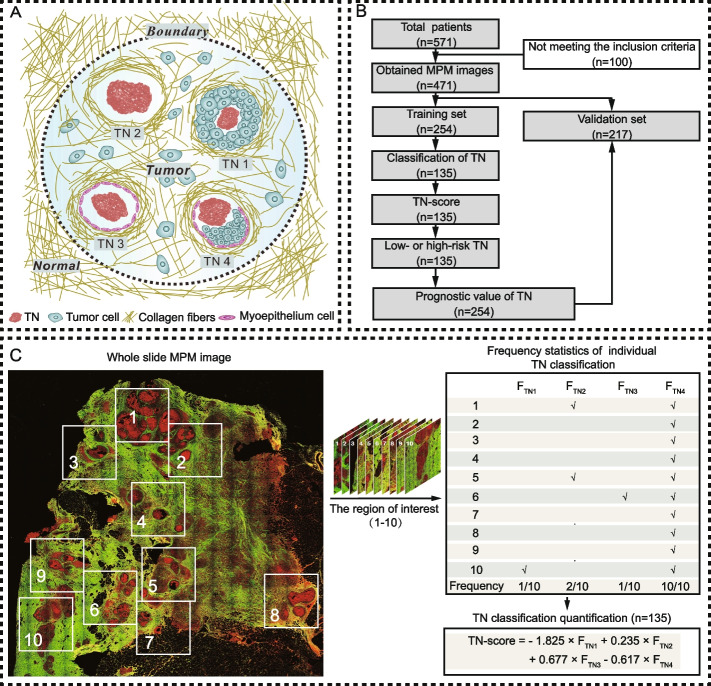
**A** Illustration of individual TN classification in the tumor microenvironment. **B** Study flow to exclude patients with neoadjuvant chemotherapy or radiotherapy, unknown pathological characteristics and follow-up, damaged and tumor-free sections, and necrosis only concentrated in an area not exceeding 2.2 mm. **C **Extraction and quantification of TNs of one patient. For this patient, a total of 10 regions of interest in the MPM image were acquired

In this study, we found that TNs also had spatial heterogeneity in breast cancer, and believed that this most easily observed feature on the histopathological examination of solid tumors might contain prognostic information that was not mined by the traditional TN classification based on presence/absence and extent. Therefore, we obtained MPM images of TN and classified TN as TN1-4 according to the relative spatial positions of TN, tumor cells, collagen fibers and myoepithelial cells (Fig. 1A). Furthermore, we creatively reflected the intratumor heterogeneity through the frequency of TN1-4. Based on the frequency of TN1-4, we obtained a TN-score for each patient and established a risk prognosis model. In addition, we also incorporated the TN-score into the current staging category to investigate the impact of TN on staging category in IBC. This is a new classification method of TN to investigate the prognosis of IBC, which is different from the traditional TN classification based on presence/absence and extent. As far as we know, this is the first time used MPM to visualize TN spatial heterogeneity and to mine the prognostic value of TN in breast cancer through frequency.

## Methods

### Patients

This study was a retrospective study conducted under the protocol approved by the Institutional Review Boards of Fujian Medical University Union Hospital and Harbin Medical University Cancer Hospital. We collected 571 formalin-fixed and paraffin-embedded tumor tissue samples from IBC patients (aged 24–87 years) who were treated in Fujian Medical University Union Hospital (Fuzhou, Southern China) and Harbin Medical University Cancer Hospital (Harbin, Northern China) from November 2003 to May 2016, in which 471 patients passed quality control for subsequent analysis (Fig. 1B). The inclusion criteria were: (1) patients had pathologically confirmed IBC without distant metastasis and underwent surgical resection; (2) there was no chemotherapy before surgery; (3) the follow-up data were complete. When the necrosis only gathered in one area less than 2.2 × 2.2 mm^2^ on H&E images, these patients were excluded. No differences were observed between the training and validation sets except for the age, tumor size, tumor grade and molecular subtype (Additional file 1: Table S1).

### Clinicopathologic characteristics and follow-up information

Clinicopathologic characteristics, including age, tumor size, nodal status, clinical stage, histological grade, and molecular subtype, were recorded. Age was determined at the time of diagnosis and classified into two categories (< 50, ≥ 50). The tumor size was recorded according to the largest diameter of the tumor in the pathological report and was classified into two categories (≤ 2 cm, > 2 cm). Nodal status was categorized as negative and positive. Clinical stage (I, II, III) of the tumor was obtained by two doctors with rich clinical experience based on clinical data. Histological grade (G1, G2, G3) of the tumor was assessed by two pathologists according to Nottingham's histologic grade. The expression of estrogen receptor (ER), progesterone receptor (PR), HER2, and the Ki67 was determined with immunohistochemical staining on formalin-fixed paraffin-embedded tissue in the pathology laboratory. A score of 3 + was defined as HER2 positive at immunohistochemical analysis, while tumors with scores of 1 + and 0 were considered to be HER2 negative. When immunohistochemical staining score was 2 + , further testing (in situ hybridization (ISH)) was needed for confirmation. The unamplified result of ISH was defined as HER2 negative, while the amplified result of ISH was defined as HER2 positive. Based on the results of immunohistochemical staining and ISH, the patients were divided into four molecular subtypes: Luminal A: ER positive and/or PR positive, HER2 negative and Ki67 low expression (< 20%); Luminal B (HER2 negative): ER positive and/or PR positive; HER2 negative and Ki67 high expression (≥ 20%); Luminal B (HER2 positive): ER-positive and/or PR positive, and HER2-positive; HER2-enriched: ER negative, PR negative and HER2 positive; Triple-negative: ER, PR and HER2 negative. Timing of recurrence, including early (within 24 months) or late recurrence (beyond 24 months), was recorded. Local recurrence was defined as any recurrence in the ipsilateral supraclavicular, infraclavicular, or axillary nodes. Median DFS follow-up was 69.0 months (IQR 32.0–81.0).

### Sample preparation and multiphoton imaging system

All the specimens were sectioned into two slices with 5 μm thickness. One was to locate all TN with H&E staining, and another deparaffinized and unstained section was to acquire MPM images and identify the classification of TN. The imaging system was built on a commercial laser-scanning microscope (LSM 880 Zeiss, Germany) using a mode-locked femtosecond Ti:sapphire laser (Chameleon Ultra, Coherent, USA) that tunable from 680 to 1080 nm operating at 810 nm [29]. The backscattered signals from tissue samples were simultaneously obtained via two independent channels. One channel detected an SHG signal (green color) between 395 and 415 nm, while the other channel detected a TPEF signal (red color) between 428 and 695 nm. MPM images were obtained by scanning unstained histological sections in the full field of vision by using a Plan-Apochromat × 10 objective (NA = 0.45, Zeiss, Germany) to determine the location of TN [29]. Areas with significantly abnormally strong TPEF signals and containing at least 10–15 adjacent, dead tumor cells were identified as the presence of TN [13]. Areas, where uncertainty existed as to whether the TPEF signal was enhanced, were coded as the absence of TN. Subsequently, the region of interest was amplified by using a Plan-Apochromat × 20 objective (NA = 0.8, Zeiss, Germany) to obtain the MPM images of TN (Fig. 1C).

### TN quantification

Throughout the entire tissue section, all non-overlapping regions of interest (ROI) with a size of about 2.2 × 2.2 mm^2^ were marked in H&E images. Next, on other deparaffinized and unstained serial sections, all corresponding labeled regions were imaged by MPM. The TN classification from MPM images was identified independently by three autonomous observers (Zhijun Li, Yu Yi, and Jianhua Chen). Each TN classification was determined as “yes” by at least two observers. After the classification of TN from MPM images was identified, the frequencies of individual TN were calculated. Subsequently, ridge regression was used to retrieve the coefficient of individual TN, and a TN-score was constructed (Fig. 1C). Each patient with TN obtained a TN-score. According to the cutoff value determined by the maximum Youden index in the training set obtained by the ROC curve analysis, they were further divided into a low-risk TN group (TN-score ≤ the cutoff value) and a high-risk TN group (TN-score > the cutoff value). Accordingly, the data set was classified into three groups: absence, low-risk TN and high-risk TN group.

To ensure reproducibility, the intraclass correlation coefficient (ICC) of the TNs determined by the three observers was calculated. We randomly selected the MPM images of 140 ROIs for TN classification to evaluate the intra-observer discordance. Each panelist performed the TN classification and repeated the same steps two days later. The mean intra-observer and inter-observer concordance of the TN classification among the three observers were 0.956 (95% CI, 0.927–0.985) and 0.851 (95% CI, 0.840–0.862). An ICC value of > 0.8 was considered highly consistent. Throughout these processes, imaging observers did not know the patient outcomes.

### Statistical analysis

Clinicopathologic variables were summarized through percentages for categorical variables, as well as medians and interquartile range (IQR) for continuous variables. Comparisons of categorical variables were evaluated using the Chi square test or Fisher’s exact test. The clinicopathologic variables with *P* < 0.05 in the univariate Cox proportional hazard model were included to identify independent risk factors using a multivariate Cox regression model. Forward stepwise regression was applied to select the independent prognostic factor. According to the TN-score, patients were classified into a low-risk TN group or a high-risk TN group by the maximum Youden index (a selected cutoff value). Disease-free survival (DFS) was calculated using the Kaplan–Meier method and the log-rank test, and hazard ratios (HR) were calculated using a univariate Cox regression analysis. To assess which model (current staging category or modified staging category) was better at stratifying patients, AUC was measured. TN-score was used as categorical variables in Kaplan–Meier curve analysis, while was used as continuous variables in the Cox regression analysis and AUC analysis. All statistical tests were two-sided, and *P* values of less than 0.05 were deemed significant. Statistical analyses were done in SPSS (version 23.0) and R (version 3.6.3).

## Results

### Definitions of TN1-4 and patient-specific quantification

**Fig. 2:**
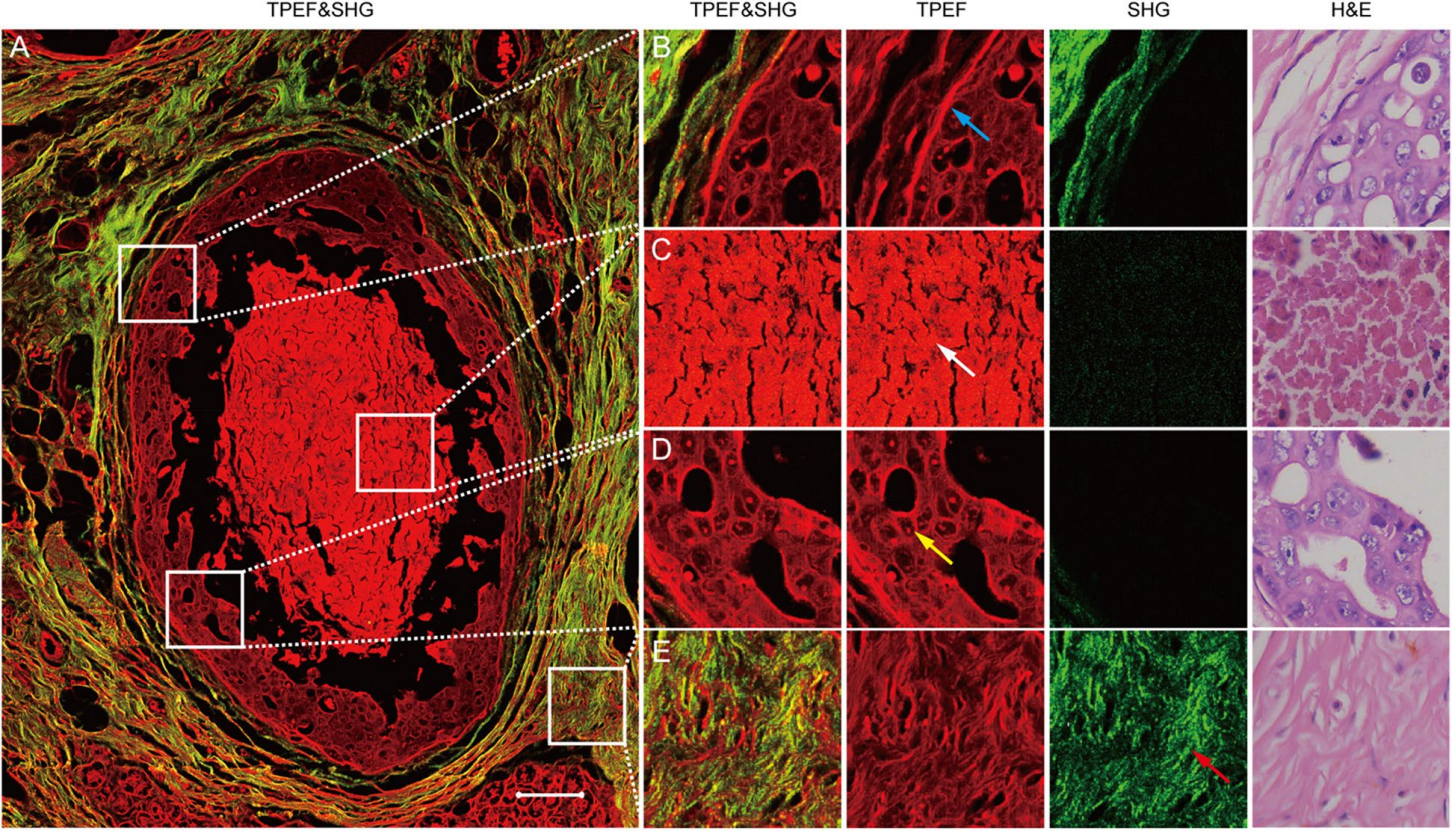
**A** MPM image of breast cancer microenvironment showing the spatial distributions of TN, tumor cells, myoepithelium and collagen fibers. **B** Magnified images showing myoepithelium in the microenvironment, which was a bright red layer in the TPEF images. **C** Magnified images showing the TN in the microenvironment, which was much brighter than tumor cells in TPEF images. **D** Magnified images showing tumor cells in the microenvironment. **E** Magnified images showing the collagen fibers in the microenvironment. Blue arrow: myoepithelium; white arrow: TN; yellow arrow: tumor cells; red arrow: collagen fibers. Scale bar: 100 μm

MPM images clearly showed the spatial distributions of tumor cells, TN, collagen fibers and myoepithelium in the tumor microenvironment (Fig. 2A). As shown in Fig. 2, myoepithelium, TN and tumor cells were detected by the signal of TPEF. The intensity of the emission spectra of TN and tumor cells was the strongest at about 468 nm. At 468 nm, the mean and standard deviation of emission intensity from TN was 580.0 ± 202.4, while from tumor cells was 181.6 ± 57.8, that was, the intensity ratio of TN over tumor cells was approximately 3 (Additional file 2: Fig. S1). Among TN, tumor cells and myoepithelium, the emission intensity of myoepithelium was the highest (800.7 ± 325.4, Additional file 2: Fig. S1), so the myoepithelium (Fig. 2B) showed a bright red layer in TPEF images. The signal intensity of TN (Fig. 2C) was significantly higher than that of tumor cells in TPEF images (Fig. 2D and Additional file 2: Fig. S1), which made the two types of cells easy to be distinguished. In addition, collagen fibers could be directly identified by SHG imaging (Fig. 2E).

**Fig. 3:**
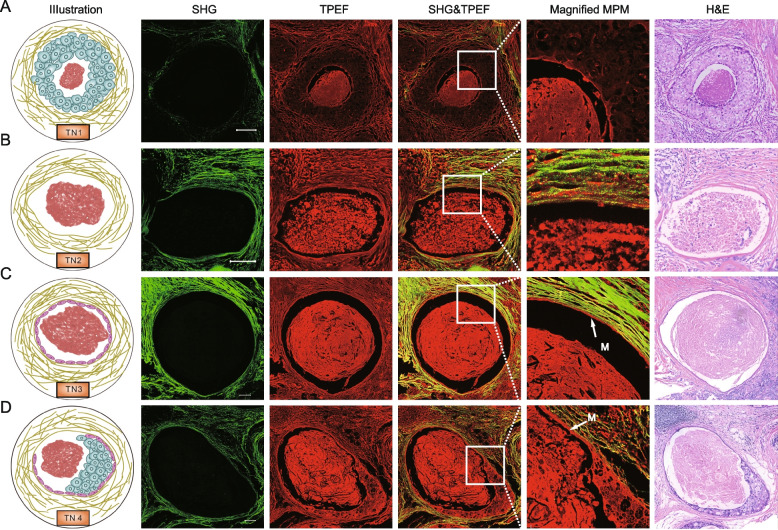
Subclassification of TN in IBC. **A** TN1, a kind of TN pattern surrounded by tumor cells and SHG images showed that tumor cells separated collagen fibers from necrotic lesions. **B** TN2, a kind of TN pattern surrounded by collagen fibers, and no tumor cells were observed between the necrotic lesions and collagen fibers. **C** TN3, a kind of TN pattern surrounded by myoepithelium, and no tumor cells were observed between necrotic lesions and myoepithelium, and SHG images showed that myoepithelium separated collagen fibers from necrotic lesions. **D** TN4, a kind of TN pattern with multiple components surrounding the necrotic lesions. M: myoepithelium. Scale bar: 100 μm

According to the relative spatial positions between TN, tumor cells, collagen fibers and myoepithelium, there were four kinds of TN (TN1-4) in the tumor microenvironment, as shown in Fig. 1A and Fig. 3. TN1 was defined as a TN in a pattern completely surrounded by tumor cells, and no collagen fibers were observed in the necrotic lesions (Fig. 3A), which included lesions surrounded by invasive breast cancer cells and lesions in duct (Additional file 2: Fig. S2). TN2 was defined as a TN in a pattern completely surrounded by collagen fibers, and no tumor cells were observed between the necrotic lesions and collagen fibers (Fig. 3B), which included lesions surrounded possibly ductal carcinoma in situ (DCIS) with regressive changes and lesions in the stroma (Additional file 2: Fig. S3). TN3 was defined as a TN in a pattern completely surrounded by myoepithelium, and no tumor cells were observed between necrotic lesions and myoepithelium (Fig. 3C). TN4 was a TN in a pattern with multiple components around the necrotic lesions (Fig. 3D), which included lesions surrounded by myoepithelium and tumor cells, lesions surrounded by collagen and tumor cells, and lesions surrounded by tumor cells but with collagen fibers (Additional file 2: Fig. S4). As shown in Fig. 1C, for a patient with TN, multiple TNs might be present in one ROI and one TN might exist in multiple ROIs. This complexity was caused by the large size (~ 2.2 mm^2^) of the ROIs. Therefore, the information on TN was quantified by the percentage of individual TN in all ROIs, and only the frequencies of TN1-4 were used to calculate the TN-score to overcome the impact of the number of different ROIs from different patients.

The patient-specific quantification was completed by quantifying TN information, age, tumor size, nodal status, clinical stage, histological grade, molecular subtype, and DFS (Additional file 1: Table S2). Based on the quantified TNs and DFS information in the training set (Additional file 1: Table S2), the R package “glmnet” was used to perform the ridge Cox regression model analysis and to retrieve the coefficients of TN1-4. Then, TN-score was calculated for each patient using the linear combination of TNs percentages weighted by their regression coefficients (Fig. 1C), which was a comprehensive impact of the 4 TNs on recurrence. TN was present in 135 (53.1%) patients and 111 (51.2%) patients in the training and validation set, respectively. Patients with TN were more likely to be patients with larger tumors and HER2-enriched breast cancer compared with patients who had no necrosis (Additional file 1: Table S3). The distribution of TN1-4 in training and validation sets was shown in Additional file 1: Table S4, and the distribution of low- and high-risk TN in training and validation sets were shown in Additional file 1: Table S3. The patients with high-risk TN were more likely to be patients with HER2-enriched and triple-negative breast cancer compared with.

### Impact of TN on 5-year DFS and recurrence

**Fig. 4:**
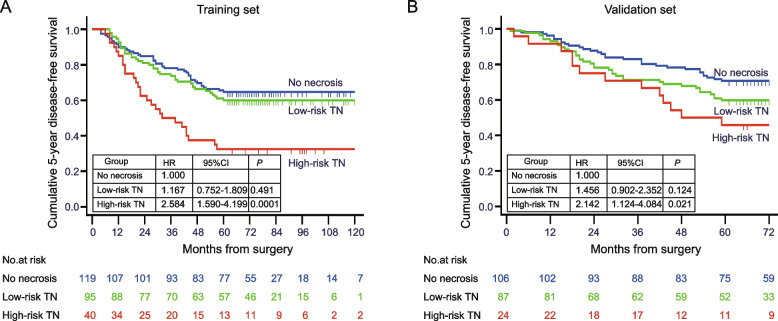
**A** 5-year DFS of patients with IBC stratified by risk of TN in the training set showed that the patients with high-risk TN had worse 5-year DFS than patients with no necrosis, while the patients with low-risk TN had a 5-year DFS comparable to patients with no necrosis. **B** 5-year DFS of patients with IBC stratified by risk of TN in the validation set also showed the same results as in the training set

In our cohorts, the patients with high-risk TN had worse 5-year DFS than patients with no necrosis (high-risk TN, 32.5% vs. no necrosis, 64.7%; *P* < 0.0001 in the training set; high-risk TN, 45.8% vs. no necrosis, 70.8%; *P* = 0.017 in the validation set). It's worth noting that the patients with low-risk TN had a 5-year DFS comparable to patients with no necrosis (low-risk TN, 60.0% vs. no necrosis, 64.7%; *P* = 0.497 in the training set; low-risk TN, 59.8% vs. no necrosis, 70.8%; *P* = 0.121 in the validation set) (Fig. 4).

A total of 116 (45.7%) and 88 (40.6%) patients had experienced a recurrence in the training and validation sets, respectively. Compared with these without necrosis, patients with necrosis were more likely to experience a recurrence (no necrosis, 37.8% vs. necrosis, 52.6%; *P* = 0.018 in the training set; no necrosis, 34.0% vs. necrosis, 46.8%; *P* = 0.053 in the validation set) (Additional file 1: Table S5), but there was no significant difference between them in the time of recurrence and the probability of distant metastasis and local recurrence. The patients with high-risk TN were more likely to experience a recurrence than patients with low-risk TN and no necrosis (high-risk TN, 70.0% vs. low-risk TN, 45.3% vs. no necrosis, 37.8%; *P* = 0.002 in the training set; high-risk TN, 58.3% vs. low-risk TN, 43.7% vs. no necrosis, 34.0%; *P* = 0.068 in the validation set). Moreover, once recurrence occurred, there was no significant difference among them in time of recurrence and the probability of local recurrence and distant metastasis.

Of note, the TN-score, along with node status, clinical stage, tumor grade and molecular subtype, were significantly associated with 5-year DFS via univariate Cox proportional hazard regression analysis (Additional file 1: Table S6, part 1). When the forward stepwise selection method was used to select independent predictors, the TN-score and clinical stage were retained, and other risk factors were excluded because they were not significantly associated with DFS in the multivariate Cox proportional hazard regression analyses (*P* > 0.05) (Additional file 1: Table S6, part 1). When all clinical factors, such as age, tumor size, nodes status, clinical stage, tumor grade, molecular subtype, chemotherapy, endocrine therapy, radiation therapy and targeted therapy were adjusted by multivariate Cox regression analysis, the TN-score remained as an independent prognostic factor for 5-year DFS (Additional file 1: Table S6, part 2 and part 3).

### Correlation of TN with tumor size and staging category

The correlation between TN and tumor size was also analyzed by comparing the percentage of TN in patients with tumor size > 2 cm and patients with tumor size ≤ 2 cm. The results showed that the incidence of TN significantly increased with tumor size (≤ 2 cm, 41.7% vs. > 2 cm, 60.9%; *P* = 0.003 in the training set; ≤ 2 cm, 44.3% vs. > 2 cm, 60.0%; *P* = 0.021 in the validation set). Among patients with tumor size ≤ 2 cm, the high-risk TN was associated with worse 5-year DFS than patients with no necrosis, while the patients with low-risk TN had a 5-year DFS comparable to patients with no necrosis in training and validation sets (Additional file 2: Fig. S5A-B). Among the patients with tumor size > 2 cm, the patients with low-risk TN had a 5-year DFS comparable to patients with no necrosis in training and validation sets. Although the high-risk TN was associated with worse 5-year DFS than patients with no necrosis in training set (Additional file 2: Fig. S5C), there was no significant difference in the validation set (Additional file 2: Fig. S5D).

**Fig. 5:**
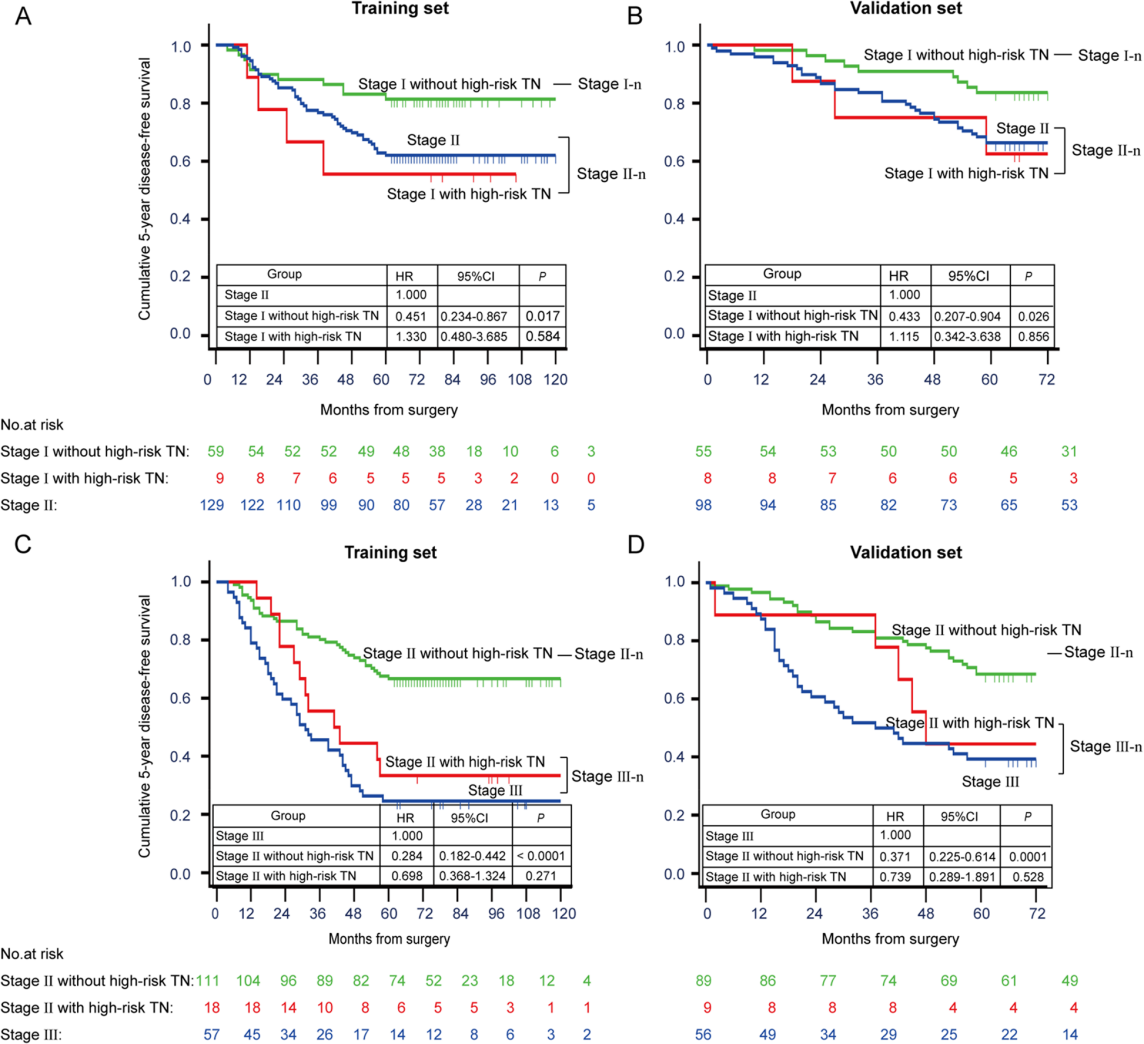
Impact of TN on 5-year DFS of patients with current pathological stage I and II tumors. **A** The pathological stage of patients who had stage I tumors and high-risk TN were up-regulated because their 5-year DFS was comparable to patients who had stage II tumors in the training set, suggesting that the patients who had high-risk TN and stage I tumors should be incorporated into stage II (stage II-n), while the patients who had low-risk TN and stage I tumors should remain in stage I (stage I-n). **B** The same results in (**A**) were validated in the validation set. **C** The pathological stage of patients who had stage II tumors and high-risk TN were up-regulated because their 5-year DFS was comparable to patients who had stage III tumors in the training set, suggesting that the patients who had high-risk TN and stage II tumors should be incorporated into stage III (stage III-n), while the patients who had low-risk TN and stage II tumors should remain in stage II (stage II-n). **D** The same results in (**C**) were validated in the validation set

Since the patients with low-risk TN had a 5-year DFS comparable to patients with no necrosis, and they all had better 5-year DFS than those with high-risk TN, we combined the patients with low-risk TN and no necrosis to analyze the impact of TN on staging category. Among the patients with stage I tumors, patients without high-risk TN had a better 5-year DFS compared with patients who had stage II tumors (81.4% vs. 62.0%; *P* = 0.014 in the training set, Fig. 5A; 83.6% vs. 66.3%; *P* = 0.021 in the validation set, Fig. 5B), but the patients with the high-risk TN had a 5-year DFS comparable to patients who had stage II tumors (55.6% vs. 62.0%; *P* = 0.565 in the training set, Fig. 5A; 62.5% vs. 66.3%; *P* = 0.856 in the validation set, Fig. 5B). Similarly, among the patients with stage II tumors, patients without high-risk TN had a better 5-year DFS compared with patients who had stage III tumors (66.7% vs. 24.6%; *P* < 0.0001 in the training set, Fig. 5C; 68.5% vs. 39.3%; *P* = 0.0001 in the validation set, Fig. 5D), but the patients with the high-risk TN had a 5-year DFS comparable to patients who had stage III tumors (33.3% vs. 24.6%; *P* = 0.271 in the training set, Fig. 5C; 44.4% vs. 39.3%; *P* = 0.519 in the validation set, Fig. 5D). The results showed that patients with high-risk TN might “up-stage” patients who had a stage I or II tumors. Therefore, the patients who had high-risk TN and stage I tumors should be incorporated into stage II, while the patients who had high-risk TN and stage II tumors should be incorporated into stage III. After incorporating TN into the staging category (i.e., I-n, II-n, III-n), patients with stage I-n versus II-n versus III-n had a higher risk of 5-year recurrence than the patients with stage I versus II versus III (Table 1). The modified staging category with incorporated TN showed higher AUC than those without incorporated TN (training set: 0.724 vs. 0.698; validation set: 0.682 vs. 0.669), indicating that the modified staging category with incorporated TN had better performance to stratify patients than the current staging category without incorporated TN.

**Table 1:** Prognostic significance of 5-year DFS in current staging category and modified staging category

Variables	Training Set		Validation Set	
	**HR (95%CI)**	***P* Value**	**HR (95%CI)**	***P* Value**
Current staging category				
I	Reference		Reference	
II	1.827 (1.024–3.258)	0.041	1.784 (0.939–3.389)	0.077
III	5.372 (2.974–9.704)	< 0.0001	4.504 (2.372–8.552)	< 0.0001
Modified staging category				
I-n (I with no high-risk TN)	Reference			
II-n (I with high-risk TN + II with no high-risk TN)	1.973 (1.014–3.839)	0.045	1.928 (0.945–3.934)	0.071
III-n (II with high-risk TN + III)	6.011 (3.136–11.523)	< 0.0001	4.953 (2.468–9.940)	< 0.0001

### Individual TN effects

To assess the individual effects of four types of TN on DFS, Spearman's rank correlation coefficient was used to analyze the correlation between individual TN and DFS. Although individual TN-specificity coefficients exhibited a small difference between training and validation sets, the relationship between TN1/2/3 and DFS was stable in the training and validation sets, that was, TN1 was consistently correlated with good prognosis, while TN2 and TN3 were consistently correlated with poor prognosis (Additional file 2: Fig. S6).

## Discussion

Precision medicine needs to accurately stratify patients according to their inherent characteristics of tumors. Several detection systems for stratified patients based on gene expression profiling have been proposed for breast cancer [31-36]. However, the problem of intratumor heterogeneity cannot be solved. Although the conventional multiregional sampling in tissue can suppress the spatial intratumor heterogeneity, the subsequent data processing is homogenized (for example, the data are routinely averaged), which results in the loss of information from intratumor heterogeneity. Since multiregional sampling is usually manifested by the diversity of morphological and spatial distributions on the digital pathological images (termed histologic patterns) [37], the solution is to directly display the information of spatial histologic patterns in situ on multiple regions [38, 39]. Recently developed space genomics technology can explore the information of spatial histologic patterns, and provide novel insights on interactions between tumor cells and nontumor cells in spatial histologic patterns [37]. However, because of the requirements of high cost and sample quality, space genomics technology has obstacles in the popularization of personalized clinical detection. Therefore, a cheaper method to show the information on spatial histologic patterns is needed in clinical applications.

As one of the most common pathological features in solid tumors, TN is easy to observe and evaluate in clinical practice. However, simple classification methods based on present/absent or extent cannot provide effective and stable prognostic information, which can be seen in a large number of conflicting reports on the prognostic value of TN. These inconsistent results may be related to the fact that traditional classification methods do not well reflect the regional heterogeneity of tumors. In this paper, we demonstrated the existence of TN spatial heterogeneity in breast cancer, which was an extrinsic manifestation of intratumor heterogeneity. Combined with this new method based on frequency estimation, the hidden prognostic value of spatial heterogeneity of TN could be partially revealed, which makes up for the shortcomings of traditional present/absent classification and extent-based classification methods. Using this new approach, we indeed find some prognostic value that is not mined by traditional present/absent classification and extent-based classification, that is, not all TNs are related to poor prognosis. TN1 was consistently correlated with good prognosis, while TN2 /3 was consistently correlated with poor prognosis. They can have different combinations in specific patient microenvironments. To show the comprehensive role of TNs and provide enough information to analyze the relationship between TN and prognosis, we quantified the frequency of individual TN in each patient and formed a TN-score. After multivariable adjustment by clinicopathologic variables, the TN-score remained a strong independent prognostic factor for 5-year DFS in the training set, which was consistent with previous studies [20, 23]. TN-score reflected the comprehensive result of the competition between the driving force promoting tumor progression and the driving force inhibiting tumor progression. When the driving force to promote tumor progression in a patient exceeded the driving force to inhibit tumor progression, the result was that the tumor continued to develop, and the patient was classified as high-risk TN group by TN-score. Conversely, when the driving force to inhibit tumor progression exceeded the driving force to promote tumor progression, the tumor development was limited, and the patient was classified as low-risk TN group by TN-score. Therefore, although there is many TN spatial histological pattern in a patient, only TN-score, a comprehensive driving force resulting in multiple regions, could predict the prognosis. After stratified patients based on TN-score, we further explored the prognostic value of TN and found that only those patients with high-risk TN were associated with poor 5-year DFS, while patients with low-risk TN had a 5-year DFS comparable to patients with no necrosis, which was not found in previous TN prognostic studies.

Previous studies suggested that the presence of necrosis was regularly associated with aggressive clinic-pathological characteristics including tumor size and ER-negative tumors. Being consistent with this result, we also found that the presence of TN was associated with larger tumors and HER2-riched tumors (Additional file 1: Table S3). What's more, we found that the high-risk TN seemed more likely to exist in ER-negative tumors, especially in triple-negative tumors. Moreover, compared with low-risk TN, high-risk TN has no preference for large tumors (Additional file 1: Table S3). It was also worth noting that patients within the same pathological stage often had different combinations of histologic patterns and different TN-scores, suggesting that the patients within the same pathological stage might also have very different prognoses. Wei et al. found that extensive tumor necrosis (≥ 50% necrosis) “up-staged” patients with hepatocellular carcinoma [6]. The same results have been shown in our work, that was, the high-risk TN “up-staged” patients with IBC. Therefore, TN-score had certain applicability in clinical stratification, which can prevent low-risk patients (such as those with low-risk TN in stage I) from being over-treated and some high-risk patients (such as those with high-risk TN in stage II) from being under treated. It should be considered to be incorporated into the current staging category, aiming at further refining patient stratification in IBC.

The results of this study have potentially important implications. First, this study reveals that the spatial histologic patterns contain information on tumor heterogeneity and prognosis. The study of prognosis should change from the conventional (spatially homogenized) biomarker to the histological patterns (spatial heterogeneity) reflecting intratumor heterogeneity. Second, the frequency of spatial histologic patterns is a method to mine information on tumor heterogeneity. The link between molecular and spatial histologic patterns remains elusive. The method of reflecting tumor heterogeneity information through the frequency of TN spatial histological patterns does not need to clarify the molecular mechanism of TN spatial heterogeneity, which has potential clinical significance. Third, although the molecular assessment of intratumor heterogeneity is still challenging, the examination of histologic patterns is convenient and low-cost, which promotes the wide application of this method in clinical applications. Fourth, this study reveals that collagen, which hitherto is ignored in pathology diagnostics for breast cancer, exhibits distinctive spatial location features in histologic patterns of TN that may inform the relevant underlying processes of breast cancer development. Studies have shown that collagen in the extracellular matrix (ECM) provides biophysical cues which can influence breast cancer progression and metastasis [40]. Collagen deposition during breast cancer progression contributes to increased matrix stiffness or elasticity, which establishes migration paths for cells. Accordingly, elevated collagen and the collagen fiber alignment relative to the breast tumor interface are risk factors for breast cancer [40]. These results indicate that the compressive stress in the tumor interior can influence the shape of the tumor mass and then influence the topographical features of the ECM, such as the formation of different spatial arrangements of collagen, which provide clues to the prognosis of breast cancer. Given accumulating evidence supporting a link between the biological properties of collagen in the tumor microenvironment and the clinical aspects of cancer [41-43], patient stratification based on relative spatial positions of TN, tumor cells, collagen fibers and myoepithelium could be clinically more relevant. Excitingly, MPM can clearly show the morphological features and spatial position of collagen in the tumor microenvironment, which is the biggest advantage of MPM but the disadvantage of H&E. If H&E is directly used to identify TN, incorrect TN classification results will be obtained (Additional file 2: Fig. S7). As shown in Additional file 2: Fig. S7, TNs with collagen and tumor cells around the necrotic lesions were classified as TN4 by MPM, while it is classified as TN1 by H&E because no collagen is observed in H&E images. Therefore, traditional H&E was not conducive to classifying TN with spatial heterogeneity. The second advantage of using MPM to identify TN was that the lesions of typical TN were much brighter than surrounding tumor cells in TPEF images, which was caused by the metabolic failure and the significant enhancement of the intensity of intrinsic fluorescence [25, 44]. Even inexperienced pathologists could easily identify TN through bright color contrast. This method of identifying TN reduces the subjectivity of empirical judgment, which provided a more objective basis for the continuous quantification of TN classifications. Thirdly, sections for MPM imaging could be obtained while H&E staining was routinely performed in the pathology laboratory, so the evaluation of clinical samples using the new method could be completed in the laboratory. Currently, MPM had been widely used in various diagnosis studies including dermatology, oncology, neurology, and cardiovascular disorders, and was expected to become a powerful clinical diagnostic tool [26, 45-48]. In 2007, König et al. reported the first clinical application of two-photon microendoscopy in detecting the extracellular matrix collagen and elastin in the dermis of patients with ulcers [49]. In 2018, Boppart et al. designed and built a custom compact and portable label-free multimodal nonlinear imaging system for intraoperative visualization of the breast tumor microenvironment [50]. Furthermore, the CE-certified in vivo femtosecond laser imaging system *DermaInspect* (JenLab GmbH) was currently in clinical use to get information on skin age, and hair pigments, and to detect dermatological disorders such as melanoma and fungal infection [51]. With the development of miniaturized equipment, MPM is expected to achieve routine clinical evaluation.

In the new TN classification method, we used the multi-mode spatial heterogeneity of TN to reflect the intratumor heterogeneity and used frequency to solve the problem of data homogenization, so as to obtain some prognostic information that was not obtained by traditional research methods. This was the innovation of this paper. It proved that hidden prognostic information could be revealed by the multi-mode spatial heterogeneity of TN. We believe that the new method might provide a new idea for the prognosis research of other malignant solid tumors.

## Conclusions

Collectively, our study demonstrates that TN-score is an independent prognostic factor in IBC. The high-risk TN is associated with poor 5-year DFS and essentially up-stage patients with IBC. Incorporating the TN-score into current staging categories can improve its performance to stratify patients.

## Supplementary Information

**Additional file 1:** **Table S1.** Characteristics of patients in the training and validation sets. **Table S2.** Quantified patient-specific data based on the training set. **Table S3.** Clinicopathological variables of patients stratified by the presence/absence classification and TN classification in training and validation set. **Table S4.** Distribution of the four types of TN in training and validation sets. **Table S5.** Recurrence patterns of patients stratified by the presence/absence classification and TN classification. **Table S6 (part 1).** Univariate and multivariate Cox regression analysis of association of risk factors with 5-year DFS in patients with necrosis in the training set (only independent prognosticators were included in multivariate analysis). **Table S6 (part 2).** Univariate and multivariate Cox regression analysis of association of risk factors with 5-year DFS in patients with necrosis in the training set (all prognosticators, except endocrine therapy and targeted therapy strongly correlated with the molecular subtype, were included in multivariate analysis). **Table S6 (part 3).** Univariate and multivariate Cox regression analysis of association of risk factors with 5-year DFS in patients with necrosis in the training set (all prognosticators were included in multivariate analysis).

**Additional file 2:** **Figure S1.** Emission spectra of myoepithelium, TN and tumor cells obtained with an excitation wavelength of 810 nm. **Figure S2.** Types of TN1. **A** Lesions surrounded by invasive breast cancer cells. **B** Lesions in duct. M: myoepithelium (white arrow). **Figure S3.** Types of TN2. **A** Lesions surrounded possibly DCIS with regressive changes. **B** Lesions in the stroma. **Figure S4.** Types of TN4. **A** Lesions surrounded by myoepithelium and tumor cells. **B** Lesions surrounded by collagen and tumor cells. **C** Lesions surrounded by tumor cells but with collagen fibers. M: myoepithelium (white arrow). **Figure S5.** 5-year DFS of patients with IBC stratified by tumor size ≤ 2 cm (**A** and **B**) and > 2 cm (**C** and **D**) in training and validation sets. **Figure S6.** Correlation analysis between individual TNs and 5-year DFS for three sets. **Figure S7.** Comparison of TN classification by MPM or H&E. In (**A**) and (**C**), TNs were classified as TN4 by MPM, because necrotic lesions were surrounded by collagen and tumor cells, while the corresponding TNs were classified as TN1 by H&E, because necrotic lesions on H&E images were only surrounded by tumor cells (**B** and **D**). White arrow: collagen, blue arrow: tumor cells.

## Data Availability

Data are available from the corresponding author on reasonable request.

## Abbreviations

AUC: Area under the curve
DCIS: Ductal carcinoma in situ
DFS: Disease-free survival
ER: Estrogen receptor
ECM: Extracellular matrix
FAD: Flavin adenine dinucleotide
H&E: Hematoxylin and eosin
HR: Hazard ratio
IBC: Invasive breast cancer
ICC: Intraclass correlation coefficient
IQR: Interquartile range
ISH: In situ hybridization
MPM: Multiphoton microscopy
NADH: Nicotinamide adenine dinucleotide
PR: Progesterone receptor
ROI: Region of interest
SHG: Second harmonic generation
TACS: Tumor-associated collagen signature
TILs: Tumor-infiltrating lymphocytes
TN: Tumor necrosis
TPEF: Two-photon excited fluorescence
