# Supplementary material for: Prognostic value of tumor necrosis based on the evaluation of frequency in invasive breast cancer
Source: BMC Cancer. 2023 Jun 9;23:530. doi: 10.1186/s12885-023-10943-x (PMC10257329; doi:10.1186/s12885-023-10943-x)
Supplement: Supplementary file 1 — Additional file 1: Table S1. Characteristics of patients in the training and validation sets. Table S2. Quantified patient-specific data based on the training set. Table S3. Clinicopathological variables of patients stratified by the presence/absence classification and TN classification in training and validation set. Table S4. Distribution of the four types of TN in training and validation sets. Table S5. Recurrence patterns of patients stratified by the presence/absence classification and TN classification. Table S6 (part 1). Univariate and multivariate Cox regression analysis of association of risk factors with 5-year DFS in patients with necrosis in the training set (only independent prognosticators were included in multivariate analysis). Table S6 (part 2). Univariate and multivariate Cox regression analysis of association of risk factors with 5-year DFS in patients with necrosis in the training set (all prognosticators, except endocrine therapy and targeted therapy strongly correlated with the molecular subtype, were included in multivariate analysis). Table S6 (part 3). Univariate and multivariate Cox regression analysis of association of risk factors with 5-year DFS in patients with necrosis in the training set (all prognosticators were included in multivariate analysis). [file 12885_2023_10943_MOESM1_ESM.doc]

ADDITIONAL FILE 1:

SUPPLEMENTARY TABLES

**Table S1.** Characteristics of patients in the training and validation sets.

| Characteristics | Training set (n=254) | Validation set (n=217) | *P* |
| --- | --- | --- | --- |
| Age (y) |  |  | 0.001 |
| <50 | 165 (65.0%) | 106 (48.8%) |  |
| ≥50 | 89 (35.0%) | 111 (51.2%) |  |
| Tumor size |  |  | 0.001 |
| ≤2cm | 103 (40.6%) | 122 (56.2%) |  |
| >2cm | 151 (59.4%) | 95 (43.8%) |  |
| Nodal status |  |  | 0.049 |
| Negative | 138 (54.3%) | 98 (45.2%) |  |
| Positive | 116 (45.7%) | 119 (54.8) |  |
| Clinical stage |  |  | 0.464 |
| I | 68 (26.8%) | 63 (29.0%) |  |
| II | 129 (50.8%) | 98 (45.2%) |  |
| III | 57 (22.4%) | 56 (25.8%) |  |
| Tumor grade |  |  | <0.0001 |
| G1 | 43 (16.9%) | 11 (5.1%) |  |
| G2 | 137 (53.9%) | 172 (79.3%) |  |
| G3 | 74 (29.1%) | 34 (15.7%) |  |
| Molecular subtype |  |  | 0.022 |
| Luminal A | 47 (18.5%) | 56 (25.8%) |  |
| Luminal B | 82 (32.3%) | 84 (38.7%) |  |
| HER2-enriched | 73 (28.7%) | 42 (19.4%) |  |
| Triple-negative | 52 (20.5%) | 35 (16.1%) |  |
| Necrosis |  |  | 0.665 |
| No | 119 (46.9%) | 106 (48.8%) |  |
| Yes | 135 (53.1%) | 111 (51.2%) |  |
| 5-years recurrence |  |  |  |
| No | 147 (57.9%) | 137 (63.1%) | 0.245 |
| Yes | 107 (42.1%) | 80 (36.9%) |  |

**Table S2.** Quantified patient-specific data based on the training set.


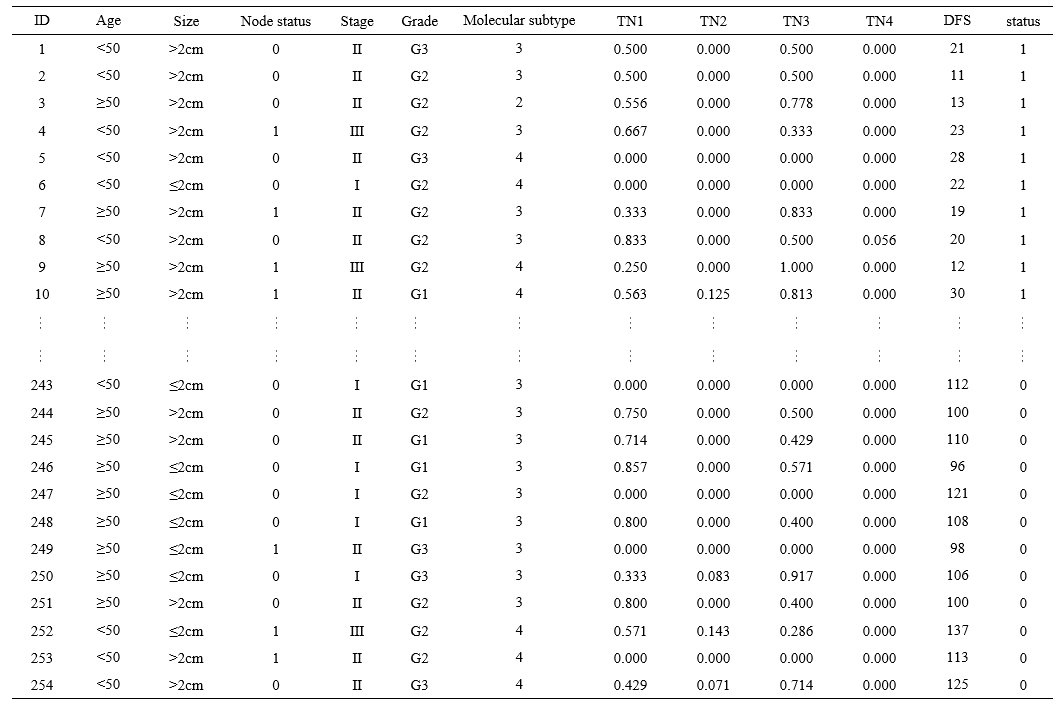


Note: ID - identification number; size: - tumor size; nodal status: 0 - negative, 1 - positive; stage - clinical stage; grade - histological grade; molecular subtype: 1- luminal A, 2 - luminal B, 3 - HER2-enriched, 4 - triple-negative; DFS - disease-free survival; status: 0 - without observed recurrence in follow-up, 1 - observed recurrence in follow-up.

**Table S3. Clinicopathological variables of patients stratified by the presence/absence classification and TN classification in training and validation set.**

| Variables | Training set | | | | | | | |  | Validation set | | | | | | | |
| --- | --- | --- | --- | --- | --- | --- | --- | --- | --- | --- | --- | --- | --- | --- | --- | --- | --- |
|  | Presence/absence classification | | |  | TN classification | | | |  | Presence/absence classification | | |  | TN classification | | | |
|  | Absence  (n=119) | Presence  (n=135) | *P* |  | Absence (n=119) | Low-risk (n=95) | High-risk (n=40) | *P* |  | Absence (n=106) | Presence (n=111) | *P* |  | Absence (n=106) | Low-risk (n=87) | High-risk (n=24) | *P* |
| Age |  |  | 0.854 |  |  |  |  | 0.166 |  |  |  | 0.952 |  |  |  |  | 0.567 |
| <50 | 78 (65.5%) | 87 (64.4%) |  |  | 78 (65.5%) | 66 (69.5%) | 21 (52.5%) |  |  | 52 (49.1%) | 54 (48.6%) |  |  | 52 (49.1%) | 40 (46.0%) | 14 (58.3%) |  |
| ≥50 | 41 (34.5%) | 48 (35.6%) |  |  | 41 (34.5%) | 29 (30.5%) | 19 (47.5%) |  |  | 54 (50.9%) | 57 (51.4%) |  |  | 54 (50.9%) | 47 (54.0%) | 10 (41.7%) |  |
| Tumor size |  |  | 0.003 |  |  |  |  | 0.007 |  |  |  | 0.021 |  |  |  |  | 0.071 |
| ≤2cm | 60 (50.4%) | 43 (31.9%) |  |  | 60 (50.4%) | 28 (29.5%) | 15 (37.5%) |  |  | 68 (64.2%) | 54 (48.6%) |  |  | 68 (64.2%) | 42 (48.3%) | 12 (50.0%) |  |
| >2cm | 59 (49.6%) | 92 (68.1%) |  |  | 59 (49.6%) | 67 (70.5%) | 25 (62.5%) |  |  | 38 (35.8%) | 57 (51.4%) |  |  | 38 (35.8%) | 45 (51.7%) | 12 (50.0%) |  |
| Nodal status |  |  | 0.734 |  |  |  |  | 0.246 |  |  |  | 0.393 |  |  |  |  | 0.499 |
| Negative | 66 (55.5%) | 72 (53.3%) |  |  | 66 (55.5%) | 55 (57.9%) | 17 (42.5%) |  |  | 51 (48.1%) | 47 (42.3%) |  |  | 51 (48.1%) | 35 (40.2%) | 12 (50.0%) |  |
| Positive | 53 (44.5%) | 63 (46.7%) |  |  | 53 (44.5%) | 40 (42.1%) | 23 (57.5%) |  |  | 55 (51.9%) | 64 (57.7%) |  |  | 55 (51.9%) | 52 (59.8%) | 12 (50.0%) |  |
| Clinical stage |  |  | 0.021 |  |  |  |  | 0.015 |  |  |  | 0.627 |  |  |  |  | 0.690 |
| I | 41 (34.4%) | 27 (20.0%) |  |  | 41 (34.4%) | 18 (18.9%) | 9 (22.5%) |  |  | 34 (32.1%) | 29 (26.1%) |  |  | 34 (32.1%) | 21 (24.1%) | 8 (33.3%) |  |
| II | 51 (42.9%) | 78 (57.8%) |  |  | 51 (42.9%) | 60 (63.2%) | 18 (45.0%) |  |  | 46 (43.4%) | 52 (46.8%) |  |  | 46 (43.4%) | 43 (49.4%) | 9 (37.5%) |  |
| III | 27 (22.7%) | 30 (22.2%) |  |  | 27 (22.7%) | 17 (17.9%) | 13 (32.5%) |  |  | 26 (24.5%) | 30 (27.0%) |  |  | 26 (24.5%) | 23 (26.4%) | 7 (29.2%) |  |
| Histological grade |  |  | 0.176 |  |  |  |  | 0.264 |  |  |  | 0.078 |  |  |  |  | 0.235 |
| G1 | 21 (17.6%) | 22 (16.3%) |  |  | 21 (17.6%) | 18 (18.9%) | 4 (10.0%) |  |  | 8 (7.5%) | 3 (2.7%) |  |  | 8 (7.5%) | 2 (2.3%) | 1 (4.2%) |  |
| G2 | 70 (58.8%) | 67 (49.6%) |  |  | 70 (58.8%) | 46 (48.4%) | 21 (52.5%) |  |  | 86 (81.1%) | 86 (77.5%) |  |  | 86 (81.1%) | 68 (78.2%) | 18 (75.0%) |  |
| G3 | 28 (23.5%) | 46 (34.1%) |  |  | 28 (23.5%) | 31 (32.6%) | 15 (37.5%) |  |  | 12 (11.3%) | 22 (19.8%) |  |  | 12 (11.3%) | 17 (19.5%) | 5 (20.8%) |  |
| Molecular subtype |  |  | <0.0001 |  |  |  |  | <0.0001 |  |  |  | <0.0001 |  |  |  |  | <0.0001 |
| Luminal A | 33 (27.7%) | 14 (10.4%) |  |  | 33 (27.7%) | 12 (12.6%) | 2 (5.0%) |  |  | 40 (37.7%) | 16 (14.4%) |  |  | 40 (37.7%) | 13 (14.9%) | 3 (12.5%) |  |
| Luminal B | 43 (36.1%) | 39 (28.9%) |  |  | 43 (36.1%) | 32 (33.7%) | 7 (17.5%) |  |  | 43 (40.6%) | 41 (36.9%) |  |  | 43 (40.6%) | 35 (40.2%) | 6 (25.0%) |  |
| HER2-enriched | 19 (16.0%) | 54 (40.0%) |  |  | 19 (16.0%) | 37 (38.9%) | 17 (42.5%) |  |  | 4 (3.8%) | 38 (34.2%) |  |  | 4 (3.8%) | 29 (33.3%) | 9 (37.5%) |  |
| Triple-negative | 24 (20.2%) | 28 (20.7%) |  |  | 24 (20.2%) | 14 (14.7%) | 14 (35.0%) |  |  | 19 (17.9%) | 16 (14.4%) |  |  | 19 (17.9%) | 10 (11.5%) | 6 (25.0%) |  |

**Table S4.** Distribution of the four types of TN in training and validation sets.

| TNs | Training set (n=135) | |  | Validation set (n=111) | |  | *P* |
| --- | --- | --- | --- | --- | --- | --- | --- |
|  | N | % |  | N | % |  |  |
| TN1 | 120 | 88.9 (120/135) |  | 101 | 90.1 (101/111) |  | 0.480 |
| TN2 | 27 | 20.0 (27/135) |  | 21 | 18.9 (21/111) |  |  |
| TN3 | 10 | 7.4 (10/135) |  | 3 | 2.7 (3/111) |  |  |
| TN4 | 129 | 95.6 (129/135) |  | 100 | 90.1 (100/111) |  |  |

**Table S5.** Recurrence patterns of patients stratified by the presence/absence classification and TN classification.

| Variables | Training set | | | | | | | |  | Validation set | | | | | | | |
| --- | --- | --- | --- | --- | --- | --- | --- | --- | --- | --- | --- | --- | --- | --- | --- | --- | --- |
|  | Presence/absence classification | | |  | TN classification | | | |  | Presence/absence classification | | |  | TN classification | | | |
|  | Absence（n=119) | Presence（n=135) | *P* |  | Absence（n=119) | Low-risk TN (n=95) | High-risk TN (n=40) | *P* |  | Absence（n=106) | Presence（n=111) | *P* |  | Absence（n=106) | Low-risk TN (n=87) | High-risk TN (n=24) | *P* |
| Recurrence | n = 119 | n = 135 | 0.018 |  | n = 119 | n = 95 | n = 40 | 0.002 |  | n = 106 | n = 111 | 0.053 |  | n = 106 | n = 87 | n = 24 | 0.068 |
| Yes | 45 (37.8%) | 71 (52.6%) |  |  | 45 (37.8%) | 43 (45.3%) | 28 (70.0%) |  |  | 36 (34.0%) | 52 (46.8%) |  |  | 36 (34.0%) | 38 (43.7%) | 14 (58.3%) |  |
| No | 74 (62.2%) | 64 (47.4%) |  |  | 74 (62.2%) | 52 (54.7%) | 12 (30.0%) |  |  | 70 (66.0%) | 59 (53.2%) |  |  | 70 (66.0%) | 49 (56.3%) | 10 (41.7%) |  |
| Timing of recurrence | n = 45 | n = 71 | 0.493 |  | n = 45 | n = 43 | n = 28 | 0.493 |  | n = 36 | n = 52 | 0.265 |  | n = 36 | n = 38 | n = 14 | 0.445 |
| Early | 18 (29.0%) | 33 (46.5%) |  |  | 18 (29.0%) | 18 (41.9%) | 15 (53.6%) |  |  | 13 (36.1%) | 25 (48.1%) |  |  | 13 (36.1%) | 19 (50.0%) | 6 (42.9%) |  |
| Late | 27 (22.7%) | 38 (53.5%) |  |  | 27 (22.7%) | 25 (58.1%) | 13 (46.4%) |  |  | 23 (63.9%) | 27 (51.9%) |  |  | 23 (63.9%) | 19 (50.0%) | 8 (57.1%) |  |
| Distant metastasis | n = 45 | n = 71 | 0.133 |  | n = 45 | n = 43 | n = 28 | 0.312 |  | n = 36 | n = 52 | 0.006 |  | n = 36 | n = 38 | n = 14 | 0.028 |
| Yes | 37 (82.2%) | 65 (91.5%) |  |  | 37 (82.2%) | 39 (90.7%) | 26 (92.9%) |  |  | 15 (41.7%) | 37 (71.2%) |  |  | 15 (41.7%) | 27 (71.1%) | 10 (71.4%) |  |
| No | 8 (17.8%) | 6 (8.5%) |  |  | 8 (17.8%) | 4 (9.3%) | 2 (7.1%) |  |  | 21 (58.3%) | 15 (28.8%) |  |  | 21 (58.3%) | 11 (28.9%) | 4 (28.6%) |  |
| Local recurrence | n = 45 | n = 71 | 0.091 |  | n = 45 | n = 43 | n = 28 | 0.221 |  | n = 36 | n = 52 | 0.441 |  | n = 36 | n = 38 | n = 14 | 0.774 |
| Yes | 15 (33.3%) | 35 (49.3%) |  |  | 15 (33.3%) | 22 (51.2%) | 13 (46.4%) |  |  | 21 (58.3%) | 26 (50.0%) |  |  | 21 (58.3%) | 19 (50.0%) | 7 (50.0%) |  |
| No | 30 (66.7%) | 36 (50.7%) |  |  | 30 (66.7%) | 21 (48.8%) | 15 (53.6%) |  |  | 15 (41.7%) | 26 (50.0%) |  |  | 15 (41.7%) | 19 (50.0%) | 7 (50.0%) |  |

**Table S6 (part 1).** Univariate and multivariate Cox regression analysis of association of risk factors with 5-year DFS in patients with necrosis in the training set (only independent prognosticators were included in multivariate analysis).

| Variable | Univariate | | |  | multivariate | | |
| --- | --- | --- | --- | --- | --- | --- | --- |
|  | HR | (95%CI) | *P* Value |  | HR | (95%CI) | *P* Value |
| Age |  |  |  |  |  |  |  |
| <50 | Reference |  |  |  |  |  |  |
| ≥50 | 1.412 | 0.863-2.309 | 0.170 |  | NA |  | NA |
| Tumor size |  |  |  |  |  |  |  |
| ≤2cm | Reference |  |  |  |  |  |  |
| >2cm | 1.311 | 0.761-2.257 | 0.329 |  | NA |  | NA |
| Nodal status |  |  |  |  |  |  |  |
| Negative | Reference |  |  |  |  |  |  |
| Positive | 2.433 | 1.473-4.018 | 0.001 |  | NA |  | NA |
| Clinical stage |  |  |  |  |  |  |  |
| I | Reference |  |  |  |  |  |  |
| II | 1.712 | 0.757-3.870 | 0.197 |  | 1.628 | 0.720-3.681 | 0.242 |
| III | 5.677 | 2.439-13.216 | <0.0001 |  | 4.787 | 2.046-11.197 | 0.0003 |
| Tumor grade |  |  |  |  |  |  |  |
| G1 | Reference |  |  |  |  |  |  |
| G2 | 2.454 | 1.033-5.827 | 0.042 |  | NA |  | NA |
| G3 | 2.396 | 0.975-5.886 | 0.057 |  | NA |  | NA |
| Molecular subtype |  |  |  |  |  |  |  |
| Luminal A | Reference |  |  |  |  |  |  |
| Luminal B | 1.711 | 0.579-5.058 | 0.331 |  | NA |  | NA |
| HER2-enriched | 1.998 | 0.695-5.743 | 0.199 |  | NA |  | NA |
| Triple-negative | 3.084 | 1.043-9.120 | 0.042 |  | NA |  | NA |
| Chemotherapy |  |  |  |  |  |  |  |
| No | Reference |  |  |  |  |  |  |
| Yes | 0.507 | 0.219-1.177 | 0.114 |  | NA |  | NA |
| Endocrine Therapy |  |  |  |  |  |  |  |
| No | Reference |  |  |  |  |  |  |
| Yes | 0.860 | 0.525-1.408 | 0.548 |  | NA |  | NA |
| Radiation Therapy |  |  |  |  |  |  |  |
| No | Reference |  |  |  |  |  |  |
| Yes | 1.247 | 0.753-2.064 | 0.391 |  | NA |  | NA |
| Targeted Therapy |  |  |  |  |  |  |  |
| No | Reference |  |  |  |  |  |  |
| Yes | 0.704 | 0.321-1.542 | 0.380 |  | NA |  | NA |
| TN-score | 3.074 | 1.849-5.111 | <0.0001 |  | 2.611 | 1.568-4.351 | 0.0002 |

Abbreviations: NA, not available

**Table S6 (part 2).** Univariate and multivariate Cox regression analysis of association of risk factors with 5-year DFS in patients with necrosis in the training set (all prognosticators, except endocrine therapy and targeted therapy strongly correlated with the molecular subtype, were included in multivariate analysis).

| Variable | Univariate | | |  | multivariate | | |
| --- | --- | --- | --- | --- | --- | --- | --- |
|  | HR | (95%CI) | *P* Value |  | HR | (95%CI) | *P* Value |
| Age |  |  |  |  |  |  |  |
| <50 vs. ≥50 | 1.412 | 0.863-2.309 | 0.170 |  | 1.322 | 0.748-2.334 | 0.337 |
| Tumor size |  |  |  |  |  |  |  |
| ≤2cm vs. >2cm | 1.311 | 0.761-2.257 | 0.329 |  | 0.890 | 0.423-1.874 | 0.759 |
| Nodal status |  |  |  |  |  |  |  |
| Negative vs. Positive | 2.433 | 1.473-4.018 | 0.001 |  | 1.400 | 0.683-2.871 | 0.358 |
| Clinical stage |  |  |  |  |  |  |  |
| II vs. I | 1.712 | 0.757-3.870 | 0.197 |  | 1.921 | 0.592-6.230 | 0.277 |
| III vs. I | 5.677 | 2.439-13.216 | <0.0001 |  | 6.090 | 1.491-24.873 | 0.012 |
| Tumor grade |  |  |  |  |  |  |  |
| G2 vs. G1 | 2.454 | 1.033-5.827 | 0.042 |  | 1.631 | 0.578-4.602 | 0.355 |
| G3 vs. G1 | 2.396 | 0.975-5.886 | 0.057 |  | 2.585 | 0.853-7.835 | 0.093 |
| Molecular subtype |  |  |  |  |  |  |  |
| Luminal B vs. Luminal A | 1.711 | 0.579-5.058 | 0.331 |  | 0.821 | 0.260-2.585 | 0.735 |
| HER2-enriched vs. Luminal A | 1.998 | 0.695-5.743 | 0.199 |  | 0.688 | 0.211-2.246 | 0.536 |
| Triple-negative vs. Luminal A | 3.084 | 1.043-9.120 | 0.042 |  | 1.212 | 0.375-3.916 | 0.748 |
| Chemotherapy |  |  |  |  |  |  |  |
| Yes vs. No | 0.507 | 0.219-1.177 | 0.114 |  | 0.252 | 0.081-0.782 | 0.017 |
| Radiation Therapy |  |  |  |  |  |  |  |
| Yes vs. No | 1.247 | 0.753-2.064 | 0.391 |  | 0.879 | 0.480-1.612 | 0.678 |
| TN-score | 3.074 | 1.849-5.111 | <0.0001 |  | 2.486 | 1.415-4.367 | 0.002 |

**Table S6 (part 3).** Univariate and multivariate Cox regression analysis of association of risk factors with 5-year DFS in patients with necrosis in the training set (all prognosticators were included in multivariate analysis).

| Variable | Univariate | | |  | multivariate | | |
| --- | --- | --- | --- | --- | --- | --- | --- |
|  | HR | (95%CI) | *P* Value |  | HR | (95%CI) | *P* Value |
| Age |  |  |  |  |  |  |  |
| <50 vs. ≥50 | 1.412 | 0.863-2.309 | 0.170 |  | 1.378 | 0.779-2.440 | 0.271 |
| Tumor size |  |  |  |  |  |  |  |
| ≤2cm vs. >2cm | 1.311 | 0.761-2.257 | 0.329 |  | 0.839 | 0.395-1.779 | 0.646 |
| Nodal status |  |  |  |  |  |  |  |
| Negative vs. Positive | 2.433 | 1.473-4.018 | 0.001 |  | 1.453 | 0.699-3.020 | 0.317 |
| Clinical stage |  |  |  |  |  |  |  |
| II vs. I | 1.712 | 0.757-3.870 | 0.197 |  | 1.946 | 0.598-6.327 | 0.269 |
| III vs. I | 5.677 | 2.439-13.216 | <0.0001 |  | 5.933 | 1.458-24.149 | 0.013 |
| Tumor grade |  |  |  |  |  |  |  |
| G2 vs. G1 | 2.454 | 1.033-5.827 | 0.042 |  | 1.668 | 0.598-4.650 | 0.328 |
| G3 vs. G1 | 2.396 | 0.975-5.886 | 0.057 |  | 2.323 | 0.760-7.107 | 0.139 |
| Molecular subtype |  |  |  |  |  |  |  |
| Luminal B vs. Luminal A | 1.711 | 0.579-5.058 | 0.331 |  | 0.809 | 0.256-2.559 | 0.718 |
| HER2-enriched vs. Luminal A | 1.998 | 0.695-5.743 | 0.199 |  | 1.652 | 0.373-7.323 | 0.509 |
| Triple-negative vs. Luminal A | 3.084 | 1.043-9.120 | 0.042 |  | 2.704 | 0.614-11.904 | 0.188 |
| Chemotherapy |  |  |  |  |  |  |  |
| Yes vs. No | 0.507 | 0.219-1.177 | 0.114 |  | 0.268 | 0.089-0.810 | 0.020 |
| Endocrine Therapy |  |  |  |  |  |  |  |
| Yes vs. No | 0.860 | 0.525-1.408 | 0.548 |  | 2.521 | 0.951-6.679 | 0.063 |
| Radiation Therapy |  |  |  |  |  |  |  |
| Yes vs. No | 1.247 | 0.753-2.064 | 0.391 |  | 0.897 | 0.479-1.681 | 0.734 |
| Targeted Therapy |  |  |  |  |  |  |  |
| Yes vs. No | 0.704 | 0.321-1.542 | 0.380 |  | 0.735 | 0.315-1.716 | 0.477 |
| TN-score | 3.074 | 1.849-5.111 | <0.0001 |  | 2.340 | 1.347-4.066 | 0.003 |
