# Supplementary material for: Prognostic value of tumor necrosis based on the evaluation of frequency in invasive breast cancer
Source: BMC Cancer. 2023 Jun 9;23:530. doi: 10.1186/s12885-023-10943-x (PMC10257329; doi:10.1186/s12885-023-10943-x)
Supplement: Supplementary file 2 — Additional file 2: Figure S1. Emission spectra of myoepithelium, TN and tumor cells obtained with an excitation wavelength of 810 nm. Figure S2. Types of TN1. A Lesions surrounded by invasive breast cancer cells. B Lesions in duct. M: myoepithelium (white arrow). Figure S3. Types of TN2. A Lesions surrounded possibly DCIS with regressive changes. B Lesions in the stroma. Figure S4. Types of TN4. A Lesions surrounded by myoepithelium and tumor cells. B Lesions surrounded by collagen and tumor cells. C Lesions surrounded by tumor cells but with collagen fibers. M: myoepithelium (white arrow). Figure S5. 5-year DFS of patients with IBC stratified by tumor size ≤ 2 cm (A and B) and > 2 cm (C and D) in training and validation sets. Figure S6. Correlation analysis between individual TNs and 5-year DFS for three sets. Figure S7. Comparison of TN classification by MPM or H&E. In (A) and (C), TNs were classified as TN4 by MPM, because necrotic lesions were surrounded by collagen and tumor cells, while the corresponding TNs were classified as TN1 by H&E, because necrotic lesions on H&E images were only surrounded by tumor cells (B and D). White arrow: collagen, blue arrow: tumor cells. [file 12885_2023_10943_MOESM2_ESM.doc]

ADDITIONAL FILE 2:

SUPPLEMENTARY FIGURES

**
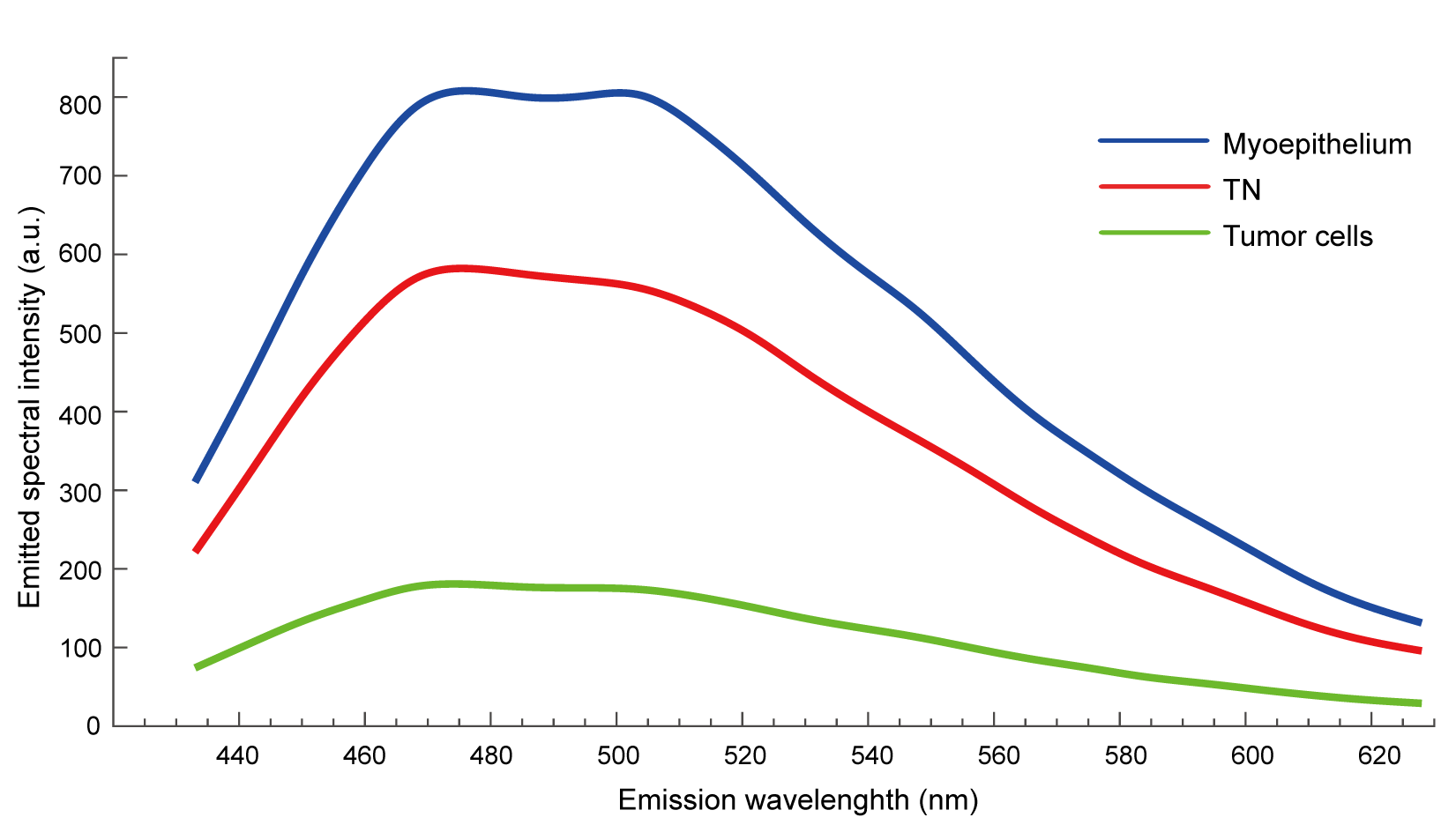
**

**Figure S1.** Emission spectra of myoepithelium, TN and tumor cells obtained with an excitation wavelength of 810 nm.

**
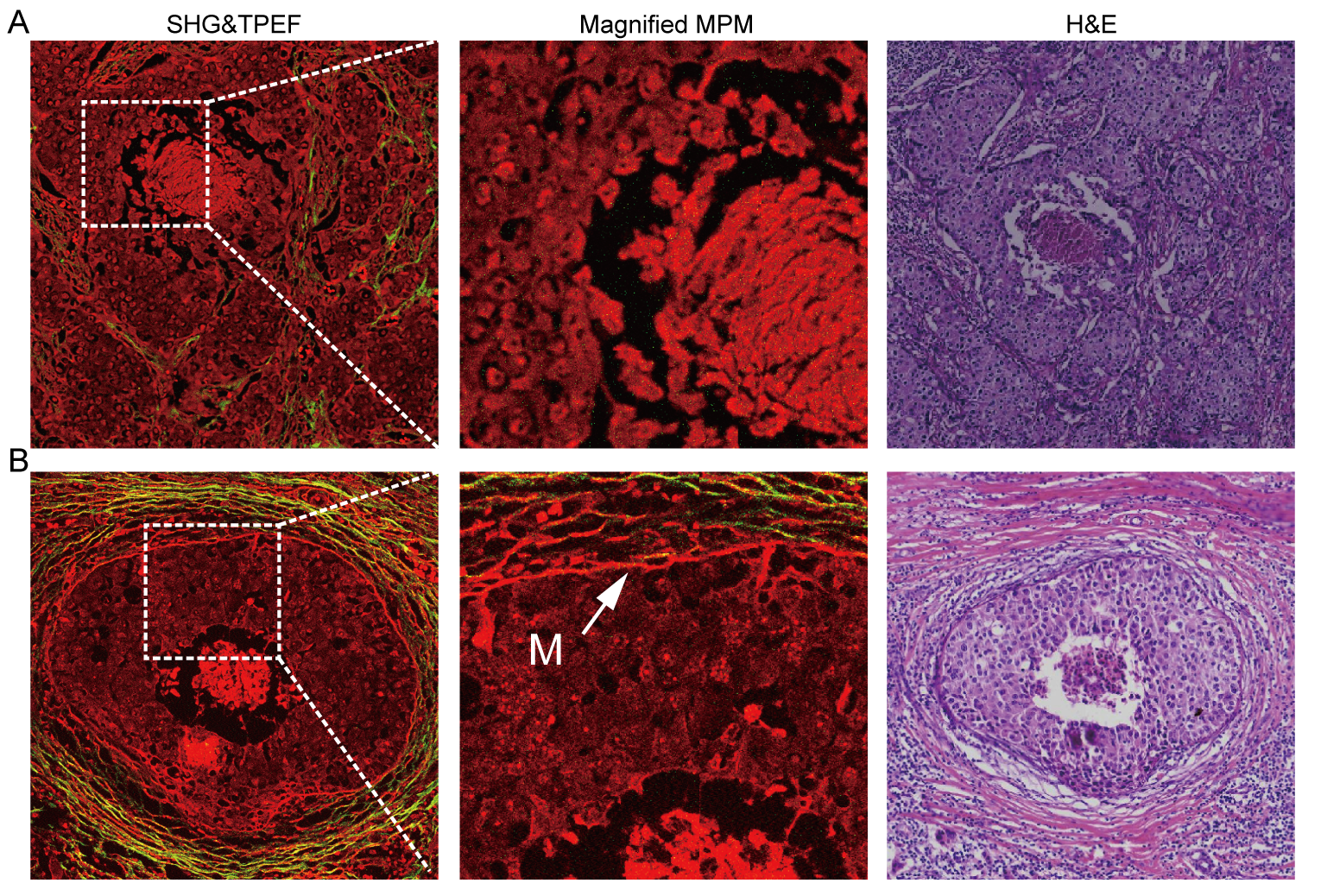
**

**Figure S2.** Types of TN1. **A** Lesions surrounded by invasive breast cancer cells. **B** Lesions in duct. M: myoepithelium (white arrow).

**
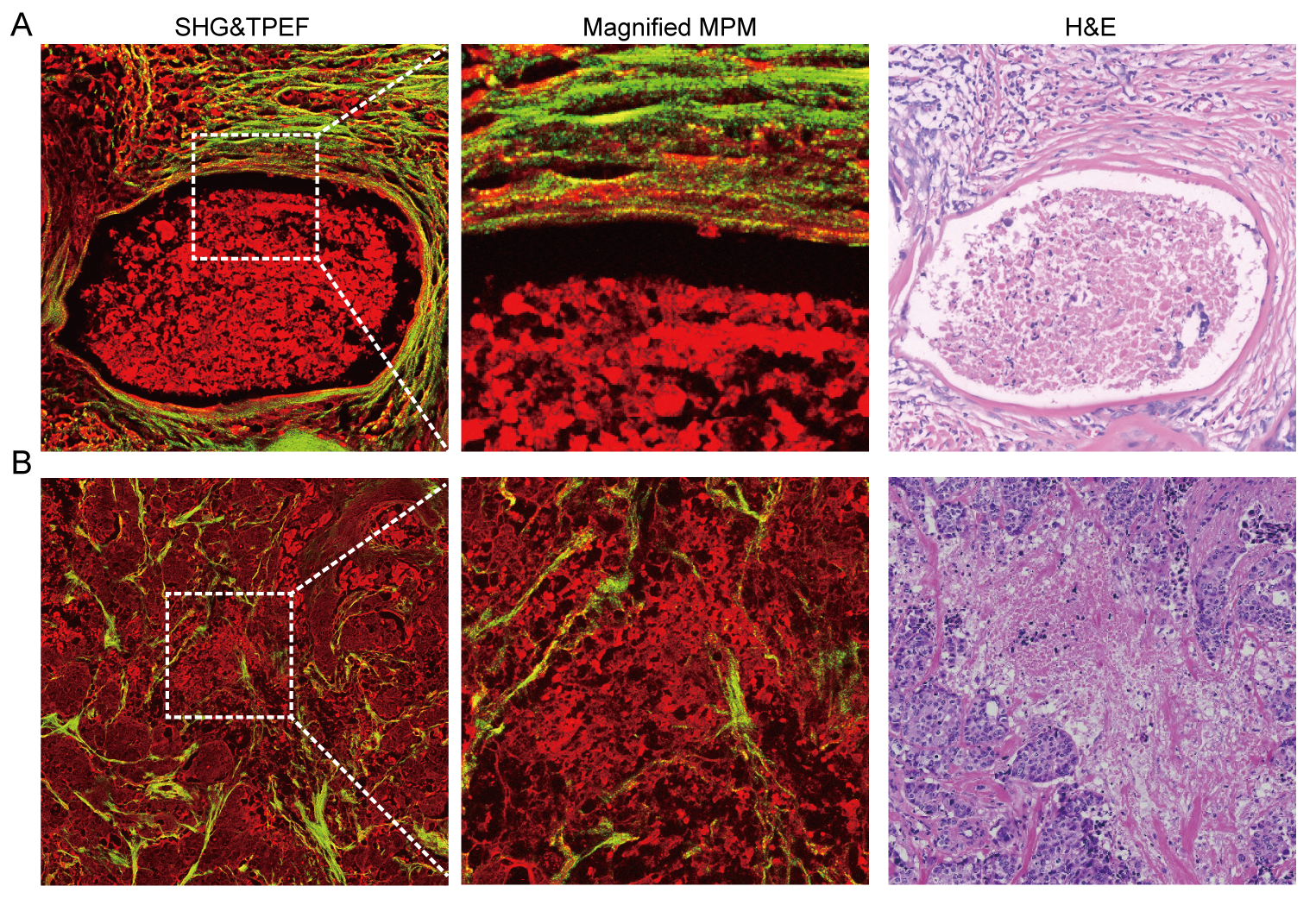
**

**Figure S3.** Types of TN2. **A** Lesions surrounded possibly DCIS with regressive changes. **B** Lesions in the stroma.


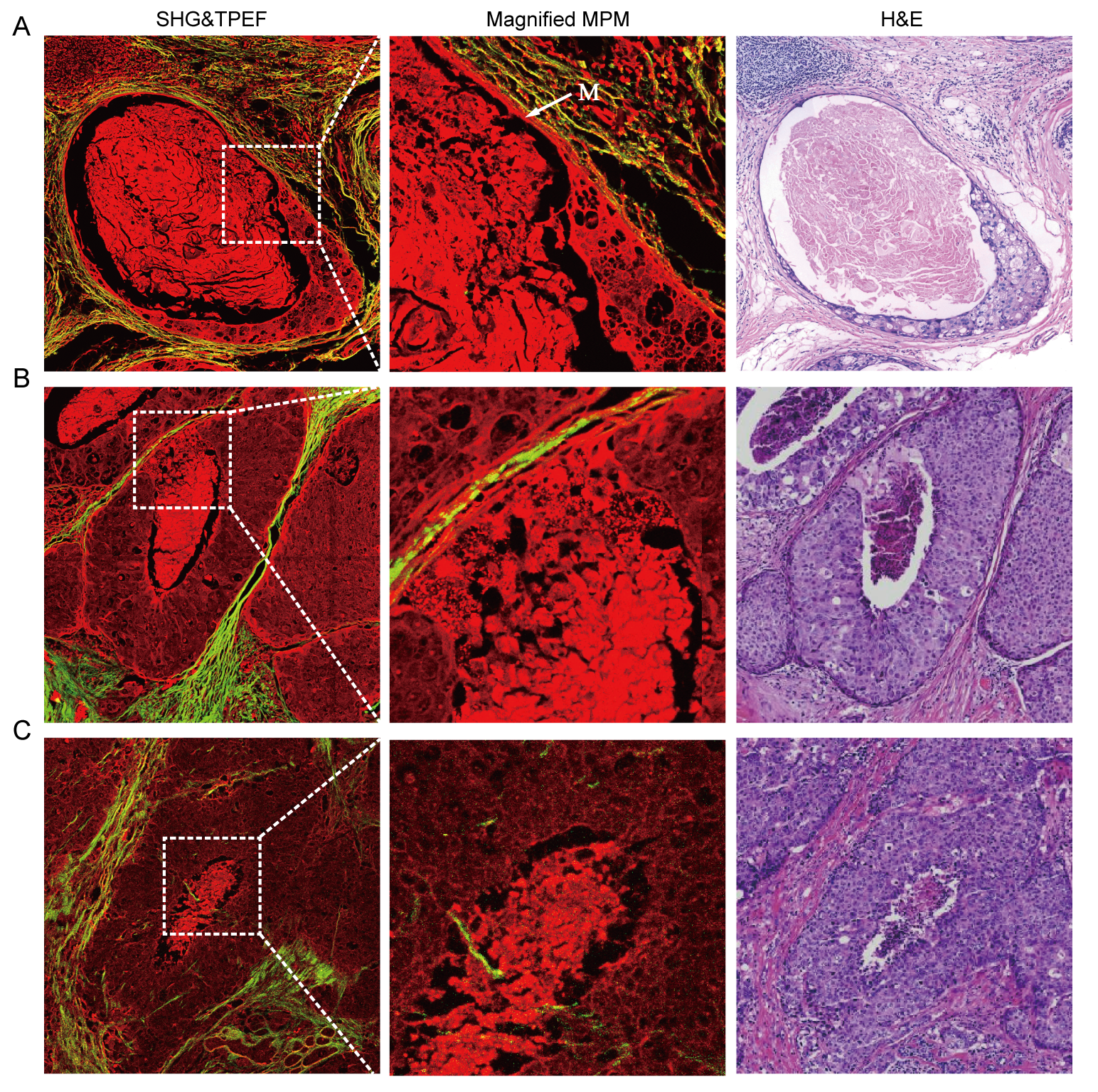


**Figure S4.** Types of TN4. **A** Lesions surrounded by myoepithelium and tumor cells. **B** Lesions surrounded by collagen and tumor cells. **C** Lesions surrounded by tumor cells but with collagen fibers. M: myoepithelium (white arrow).


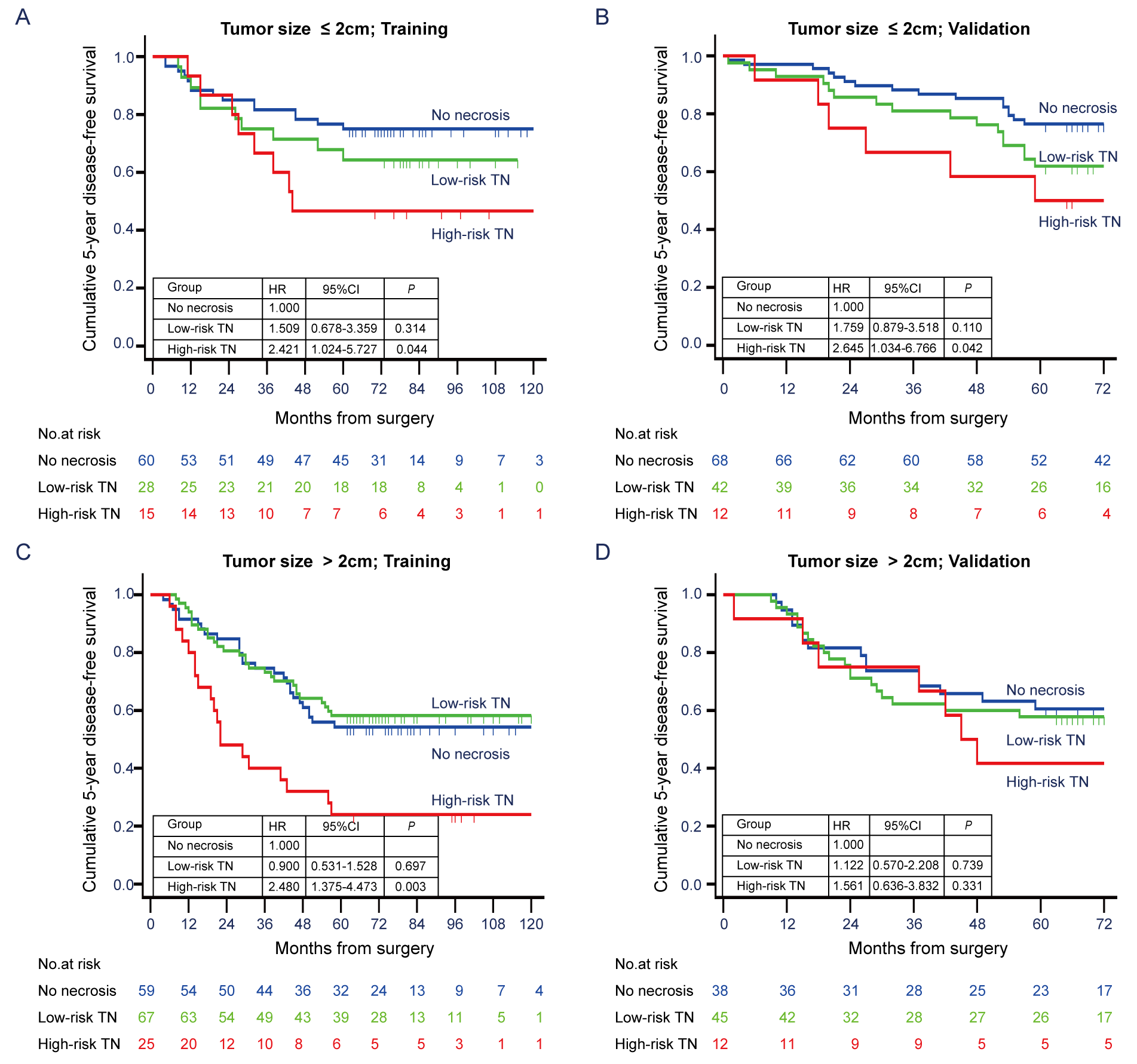


**Figure S5.** 5-year DFS of patients with IBC stratified by tumor size ≤ 2 cm (**A** and **B**) and > 2 cm (**C** and **D**) in training and validation sets.

**
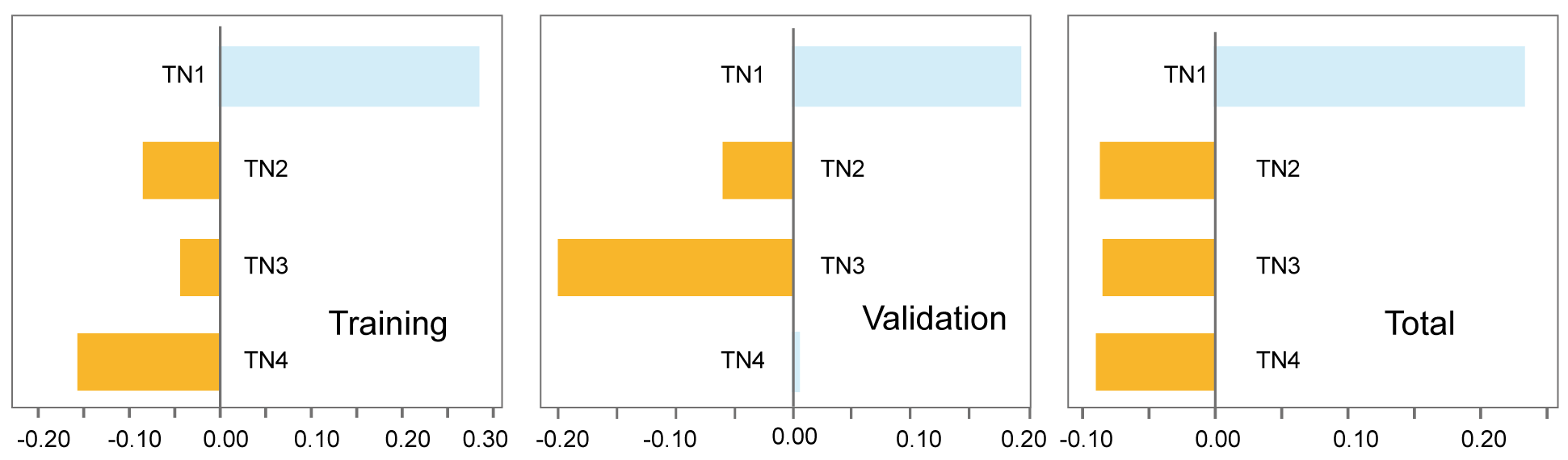
**

**Figure S6.** Correlation analysis between individual TNs and 5-year DFS for three sets.

**
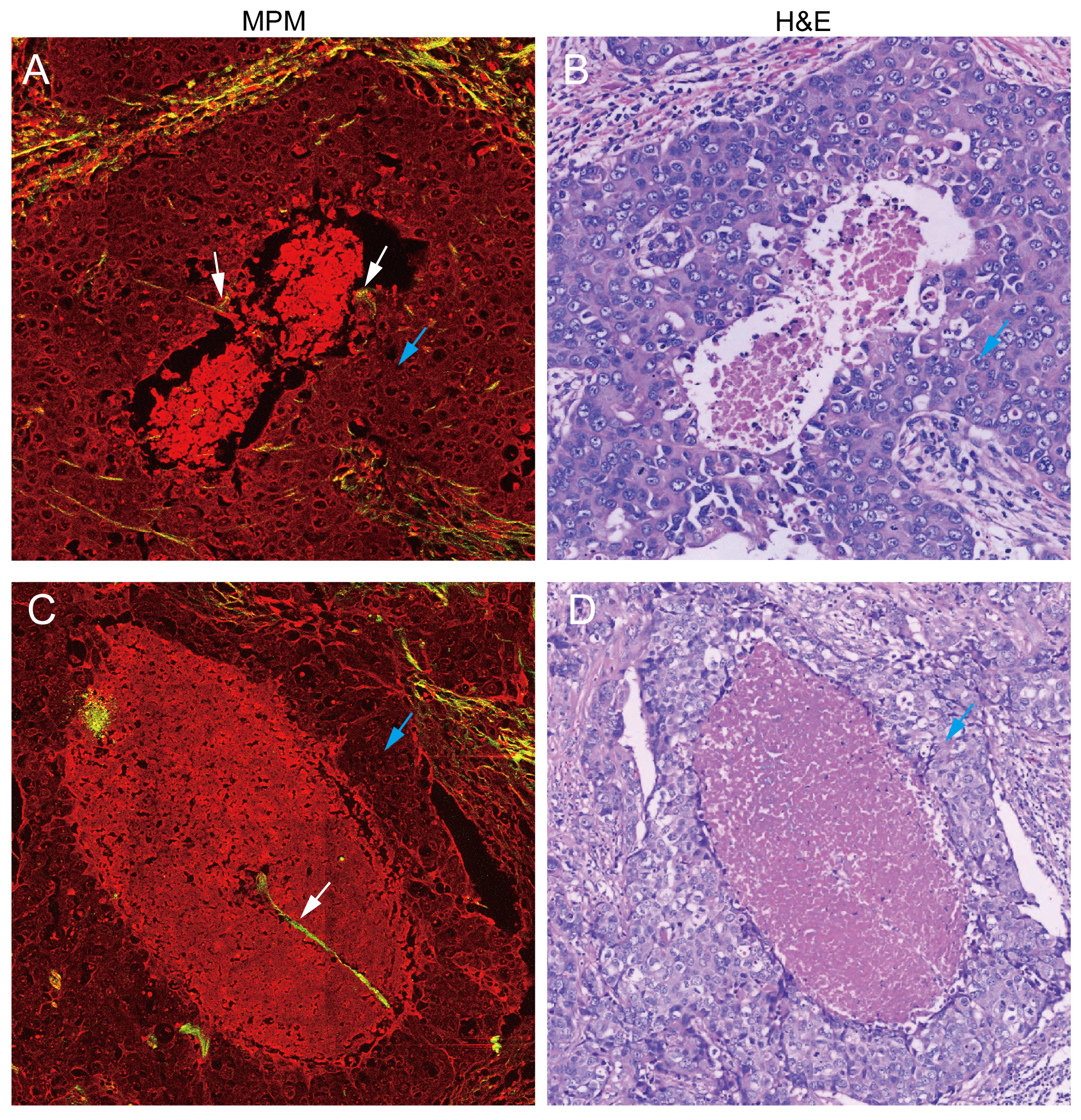
**

**Figure S7.** Comparison of TN classification by MPM or H&E. In (**A**) and (**C**), TNs were classified as TN4 by MPM, because necrotic lesions were surrounded by collagen and tumor cells, while the corresponding TNs were classified as TN1 by H&E, because necrotic lesions on H&E images were only surrounded by tumor cells (**B** and **D**). White arrow: collagen, blue arrow: tumor cells.
